# An Analysis of Global Research Trends on Greenhouse Technology: Towards a Sustainable Agriculture

**DOI:** 10.3390/ijerph17020664

**Published:** 2020-01-20

**Authors:** José A. Aznar-Sánchez, Juan F. Velasco-Muñoz, Belén López-Felices, Isabel M. Román-Sánchez

**Affiliations:** Department of Economy and Business, Research Centre on Mediterranean Intensive Agrosystems and Agrifood Biotechnology, University of Almería, 04120 Almería, Spain; jfvelasco@ual.es (J.F.V.-M.); blopezfelices@ual.es (B.L.-F.); iroman@ual.es (I.M.R.-S.)

**Keywords:** greenhouse, technology, irrigation, soil, energy, structure, pest, climatic control, sustainability, bibliometric analysis

## Abstract

Greenhouse farming is an agricultural management system that has demonstrated its efficiency in intensifying food production. These systems constitute a feasible alternative for ensuring food supply, which is one of the greatest challenges faced by humankind in the twenty-first century. Technology has been able to meet the challenges related to greenhouse farming in both contributing to overcoming its limitations, correcting adverse impacts and ensuring system sustainability. The objective of this article is to analyse the global research trends in greenhouse technology over the last two decades, in order to identify the main driving agents, the most outstanding research lines and possible gaps in the literature. Different methodologies have been used for the analysis; both quantitative and qualitative. The principal results show that there are different relevant lines of research related to different aspects of greenhouse farming: the use of water for irrigation, the design of the optimum structure of the greenhouse, conserving the soil in the best growing conditions, energy consumption of the system as a whole, climate control within the facility and pest control. The research is characterized by the being composed largely of ad hoc studies, which hinders the international collaboration between researchers and institutions. The research approach has shifted from being focused on increasing production and cost savings to aspects related to resource conservation and sustainability.

## 1. Introduction

The current growth trend of the human population, together with the evolution of consumption patterns, increasing demand and food waste are placing unprecedented pressure on agricultural systems and natural resources [1]. Therefore, the supply of food is one of the greatest challenges that humankind must face in the twenty-first century [2]. Agricultural ecosystems are the principal providers of food. Currently, approximately 275 million hectares are dedicated to irrigated crops throughout the world [3]. This area is growing at an average annual rate of 1.3% [4]. This accounts for just 23% of farmed land; however, 45% of total food production is obtained through these types of crops [5,6]. In order to satisfy the food demand in 2050, world production must increase by 70% [7]. This predicted increase in world food production implies an extension of farmed land or an intensification of the production on the land currently farmed [8]. In low production scenarios, an increase of 53% in the consumption of water resources would be required together with an increase of 38% of farmed land throughout the world in order to satisfy the food demand target in 2050 [9,10]. The main disadvantage of expanding agriculture is the availability of land, obliging the transformation of land uses, which translates into a loss of natural ecosystems [11]. Therefore, converting land into farmland is the second greatest global threat to preserving biodiversity, due to the deforestation processes involved [12].

Within this context, some authors use the “sustainability” concept to find a solution for the posed challenge. The term “sustainability”, as it is today known, was borne in 1987 within the Brundtland Report by the United Nations World Commission on Environment and Development [11]. In this report, sustainable development is defined as the one that meets current needs without compromising the ability of future generations to meet their needs [13]. Sustainability embraces three dimensions of the human and natural systems: social, environmental and economic. It remains as an ideal similar to democracy, justice and freedom [14]. After this report, some relevant events have brought about new treaties advocating the sustainable development of the Earth, like the Rio Declaration in 1992 [15], under which biologic natural resources of the planet are protected and a sustainable use of them is promoted. Furthermore, the Kyoto Protocol [16] binds the signing countries to reduce their greenhouse gas emissions, and the United Nations Millennium Development Goals [17] gave orientations to improve globally life means and the environment.

Nowadays, Sustainability Sciences can be defined as a discipline that studies the ways to reach a sustainable society through the understanding of interactions among nature, humans and society. It brings together a great variety of academic disciplines [18,19,20]. At the end of the 90s, sustainability was linked to ecosystems and their ability to keep their delivered service flows in environmental, economic and social contexts. It also relates to the development of agriculture and to a sustainable use of water [21,22]. The goals of a sustainable management in agriculture consider the agricultural system continuity from a physical and a biological perspective. They also pursue economic efficiency in the use of resources and social participation in the decision-making processes [23,24]. The increase in agricultural production, maintaining the balance between production and the preservation of nature, has become a key challenge for the sustainable management of the planet in the future [25,26,27]. In this context, a new concept has emerged within the sustainability field which has been termed as “Sustainable intensification”. Pretty et al. [28] defined sustainable intensification as the production increase on the same land at the time that negative environmental impacts are being reduced. Contributions to natural capital and the flow of environmental services increase as well. The term “Sustainable intensification” started to be used in 2008 with FAO Declaration about the need to increase food production in order to meet the nutrition needs of a growing population, which would reach 9.000 million in 2050 [29]. Under this new term it is understood that the main objective to be pursued is the increase of food supply with lower (or without) pressure on the natural environment [30]. The sustainable intensification of agriculture allows meeting the objective of increasing food production and the one of achieving a sustainable development [31]. Tilman et al. [32] estimate that food demand in 2050 will increase 100–110% in the 2005–2050 period, according to the caloric content of crops. If a moderate intensification of already in-use farming land takes place in order to meet this demand, it is foreseen that the farming land should be extended in 200 million hectares. In the case of agricultural extensification, the additional required surface would amount to 2010 million. The agricultural intensification should base on technological improvements, adaptation and transfers of intensive farming technologies to the non-intensive agriculture. In this sense, the efficient management practices could considerably reduce the use of nitrogen and the emission of greenhouse gases.

Sustainable intensification is a goal, although there is no a universal recipe to achieve it. The merits of various approaches like those related to high technology and organic agriculture should be rigorously studied and assessed taking into account biophysical and social parameters [12]. In this way, the intensification of production on the current land area used for agriculture is a promising alternative and greenhouse farming is one of the most relevant options. In recent decades, the intensification of greenhouse farming has increased production and satisfied the growing demand for resource-intensive diets [32,33]. Furthermore, industrial agricultural activities also help to generate employment, contributing to economic growth and drive the services sector in industrial regions [34]. Therefore, greenhouse agriculture has expanded rapidly in many regions throughout the world in recent decades [35]. On the other hand, this type of agriculture is not exempt from limitations and disadvantages. Water scarcity, nutrient-depleted soils and pollution constitute some of the major challenges in intensive agriculture [36]. The intensification of agriculture is achieved by applying high levels of inputs such as fertilizers or herbicides, which can contaminate the environment and affect the health of the local populations [37,38]. In many studies, the use of these types of inputs and certain practices related to intensive agriculture have been associated to the loss of biodiversity and the reduction in the flow of ecosystemic services, placing the maintenance of human well-being at risk [39]. Furthermore, agricultural overexploitation can affect the fertility and erosion of the soil if the practices do not preserve its characteristics [40,41]. Many researchers indicate that, in order to fulfil the objective of food safety in a sustainable way, food production should be increased substantially while, at the same time the ecological footprint of agriculture is reduced drastically [32,42]. According to certain authors, despite the urgency of addressing this global problem, it is receiving little attention from the scientific community or the political sphere, given the lack of objectives in quantitative terms [43,44].

However, technological development provides the fundamental elements to take on these challenges and to contribute to achieving a greater sustainability in agriculture, both in terms of resource depletion and socio-economic and environmental dimensions [45,46]. The greenhouse industry is continually developing new strategies and technologies to resolve specific limitations of the crops, reducing any related environmental impact and adapting to the new market requirements [35]. Therefore, soilless crops [36]; the comprehensive control of the factors that constitute the microclimate inside the greenhouse [47,48]; the creation of vertical agroecosystems that can be located in urban environments [49]; the development of specific environmentally sustainable solutions for supplying greenhouse production with renewable energy [50,51]; the development of new materials and structures capable of optimising production [52,53]; etc. are all now possible. Furthermore, technology has enabled the diversification of water sources for irrigation where this resource is the principal limiting factor, such as the use of reused water, desalinated seawater or rainwater collection [36,54,55,56]. 

All of these innovations have given rise to a significant increase in the studies published on greenhouse technology in recent years. However, there are no studies that have analysed all of this research as a whole. The objective of this article is to identify the main driving agents, the most outstanding research lines and possible gaps in the literature regarding greenhouse technology research. The questions which it attempts to respond to are: What are the principal driving agents of the research? What are the most representative research lines? What are the most prominent fields of technological innovation? What are the research needs? In order to respond to these questions, a quantitative and qualitative analysis of the research carried out on a global level over the last two decades has been conducted.

## 2. Methodology

### 2.1. Bibliometric Analysis

In order to meet the pursued research objective and to answer the proposed questions, the applied research methodology is the bibliometric analysis. The bibliometric analysis was developed by Garfield during the 50s in order to identify, organize and analyse the main elements within a knowledge field [57,58]. After the first works, this methodology has been extended to many disciplines like medicine, biology, ecology, engineering, administration and economics [59]. This methodology applies mathematic and statistic tools in order to determine trends, identify the main driving agents and the publication relevance within a field of study [60,61]. In the bibliometric analysis, indicators related to quantity, relevance and structure can be distinguished [62]. Quantity indicators refer to the volume of published works and the agents’ productivity (authors, journals and institutions) in a specific field. Relevance indicators show the impact of publications. Under these indicators we can find the number of quotations, the H-Index and journal classifications. Structure indicators analyse the established networks among the different elements of a research field. Traditionally, three main approaches to develop a bibliometric work can be distinguished: co-occurrence, co-citations and coupling analyses [63]. In our study, we have followed a traditional approach based on co-ocurrence. Fort he studied analysis, we have included productivity indicators, quality and structure indicators [64]. 

Firstly, agents –authors, institutions, countries and journals- which publish at most are identified. Secondly, the impact of publications by agent is analysed. This analysis, especially when related to journals, is of great interest for researchers since it means a method to assess the journal relevance where they are publishing their works [65,66]. The selected indicators are the count of quotations, the h index and the impact on the SCImago Journal Rank. The h index is defined as the total h of N documents with at least h cites each, whereas the other articles have h cites each. In means, the h index for one author or country is the h number of documents by author or country with at least h cites each [67]. The SJR measures the weight cites received by the journal, where the cite weight depends on the studied field and the prestige of the quoted journal [64]. Thirdly, we apply maping techniques to analyse the network structure among agents.

### 2.2. Data Processing

The Elsevier Scopus database has been selected to obtain the sample of studies to analyse, in the same way as other articles that have analysed the dynamics followed by a certain line of research [68,69,70,71]. The search parameters selected include all the terms related to the principal topics of the research in greenhouse technology (GT), such as soil, energy, structure, climate, and irrigation. These parameters were used in the search fields of title, abstract and keywords. The search was carried out in June 2019. The study period selected was 1999 to 2018. Only documents until 2018 have been included so that complete annual periods can be compared [72]. In addition, a search of articles on “agriculture” was also carried out with the same restrictions in order to analyse the relative importance of the research in GT within this general theme. Given that the results of the same study are frequently published as conference papers, book chapters and articles, in order to avoid duplications, only original articles have been included in the sample [73,74]. The final sample analysed is composed of 706 articles. The articles from the search were evaluated and classified depending on the number of articles, their year of publication, the language of the publication, all of the authors of the articles, the institutions and countries of all of the authors, the subject area in which Scopus classifies the studies, the name of the journals in which they have been published and the keywords. 

**Figure 1:**
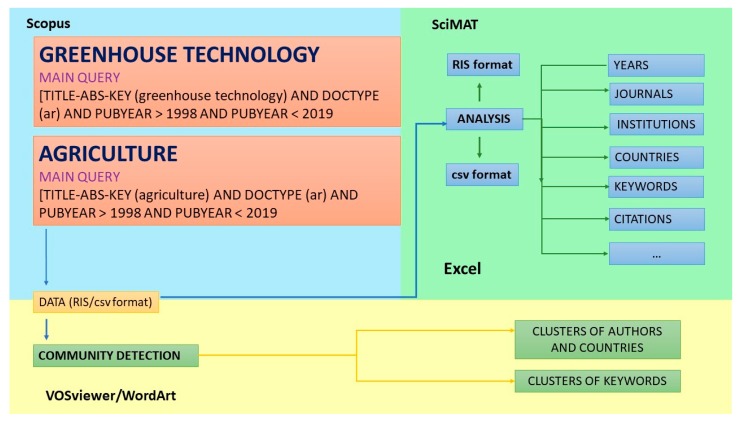
Methodology flow chart.

The data were refined to eliminate duplications, omissions and errors and to search for incomplete information. The records obtained have been analysed and the results have been grouped in tables and represented in graphs in order to show the results clearly. Finally, the keywords analysis was used to extract the principle research trends. The list of the most-used keywords in the documents comprising the sample has been analysed both qualitatively and quantitatively. The different tools used for the processing and subsequent analysis of the data were Excel (version 2016), SciMAT (v1.1.04, University of Granada, Granada, Spain) and VOSviewer (University of Leiden, Leiden, Netherlands), which are commonly used for this type of study [19]. The free software WordArt (https://wordart.com/) was used to represent the complete series and groups of keywords through word clouds [75]. Figure 1 shows the methodological approach followed in the present work. 

## 3. Results

### 3.1. The Evolution over Time of the Principal Variables Analysed 

**Figure 2:**
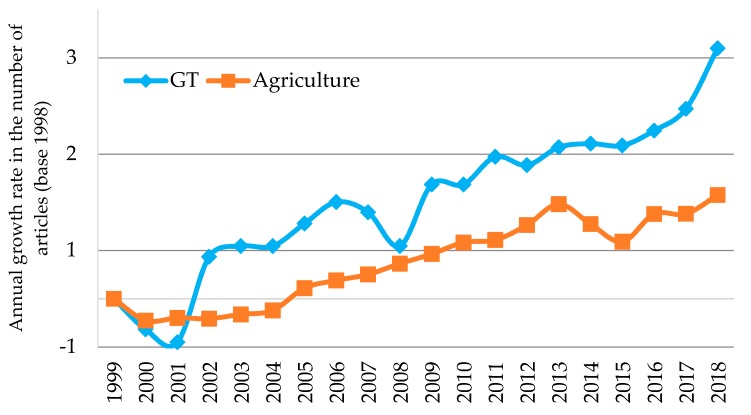
Comparative trends in greenhouse technology (GT) and agriculture research.

Table 1 shows the evolution of the research in greenhouse technology (GT) during the period 1999–2018 in terms of the number of articles published, the number of authors, the number of journals in which the studies have been published, the number of countries that have participated in the research, the total number of citations and the average number of citations per article. This group of variables has grown progressively over the whole period of study. However, the most intense development in the number of articles is concentrated in the last five years, with 45% of the total articles of the sample analysed being published during these years. In 1999, eleven articles were published on GT, while in 2018 this figure had risen to 67. In order to contextualise the development experienced by this line of research, we have compared the variation trends in the number of articles published on GT with that of the number of articles published on agriculture in general. To do this, the annual percentage variation in the number of articles published in both lines of research has been calculated, with the year 1999 taken as a base. Figure 2 shows a graphic representation of the results. The number of articles on agriculture has increased at an average annual rate of 0.4% throughout the period while the articles on GT have grown by 1.1%. Therefore, it may be concluded that GT is a line which as gained relevance in the research on agriculture. 

During the whole period analysed, a total of 2310 authors have participated in the 708 articles that make up the sample. This is the variable that has experienced most growth, increasing from 32 authors in 1999 to 319 in 2018. The average number of authors per article has doubled from 2 to 4. At the beginning of the period, the eleven articles of the sample were published in ten different journals. In 2018, the 67 articles analysed were published in 51 journals. The average number of articles per journal has remained constant over the whole period at around one. The number of countries involved in the research in GT has experienced a more moderate variation. While in 1999 there were eleven (Australia, Belgium, Denmark, Greece, Israel, Japan, Kenya, Netherlands, Spain, USA, and Uzbekistan), in 2018 there was a total of 27. The number of citations obtained per article has grown from 0.1 in 1999 to 12.1 in 2018. 

### 3.2. Evolution of Research in GT by Subject Area

**Figure 3:**
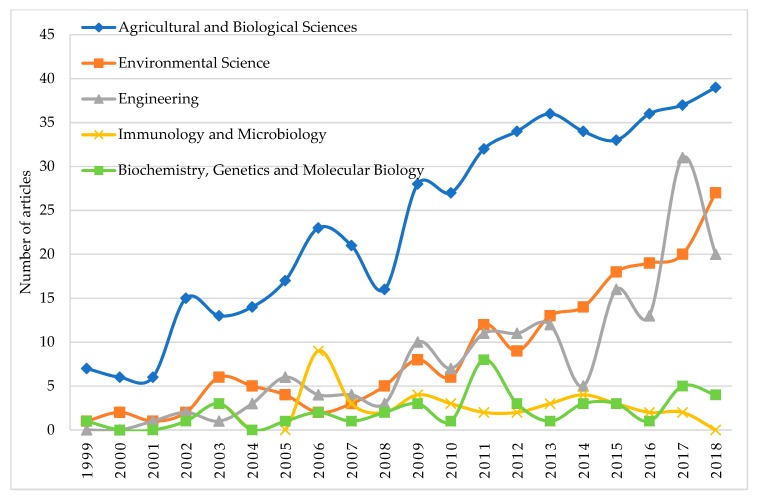
Comparative trends of subject categories related to GT research.

Figure 3 shows the evolution of the number of articles published, classified by discipline. Over the whole period analysed, the principal subject categories were Agriculture and Biological Sciences which accumulated 90.3% of the total, Environmental Sciences with 33.7%, and Engineering with 30.5%: the rest of the categories account for less than 10% of the studies in the sample. It should be noted that the same article may be classified in more than one category simultaneously. It may be observed that the research in GT is dominated by technical disciplines. In fact, only 3.6% of the articles fall within the Social Sciences category, while Economics, Econometrics and Finance account for just 0.8%. These low percentages reveal the lack of studies within the research in GT from these areas of knowledge.

### 3.3. Most Relevant Journals in Research on GT 

The journals that have published the highest number of articles on GT are included in Table 2. It shows the principal characteristics of the journals such as the country, its impact factor and its quartile position in the Scimago Journal Rank (SJR) ranking. It also includes the characteristics of the articles published on GT such as their number, their H index, the total number of accumulated and average citations and the period of publication. Overall, these journals account for 183 articles of the total sample analysed, which represents 25.9%. Of the total 310 journals in which the studies of the sample have been published, only 3.2% of them have published at least 10 articles. This indicates that there is a wide dispersion of the journals that publish articles on this subject. This could represent a difficulty when searching for information or selecting a journal in which to publish studies on GT.

The journal with the highest number of articles published on GT is Nongye Gongcheng Xuebao Transactions of the Chinese Society of Agricultural Engineering, with a total of 49. This Chinese journal has an H index of 10, a total number of citations of 309 and its average number of citations per article is 6.3. It has an SJR impact factor of 0.422, which places it in the second quartile in the category of Agricultural and Biological Sciences. It published its first article on GT in 2005 and continues to publish on this subject matter. Acta Horticulturae is the journal in second position with respect to the number of articles published throughout the period, with a total of 30. This Belgian journal is also the oldest of the group, with its first article published in 1999, still publishing on the subject today. It has an H index of 5, a total of 49 citations and an average of 1.6 citations per article. It has an SJR impact factor of 0.185, which places it in the fourth quartile in the category of Agricultural and Biological Sciences. In third position in terms of the number of articles is the British journal, the International Journal of Systematic and Evolutionary Microbiology with 17 articles. The period of publication of these articles is 2006 to 2016. This journal, together with Biosystems Engineering holds the first position in terms of its H index which is 11. The 17 articles accumulate a total of 327 citations which is an average of 19.2 citations per article. Its impact factor is 0.912 and it is in the first quartile of the SJR. 

The journal with the highest total number of citations is Biological Control with 444. This American journal also has the highest average number of citations per article, with 34.2. The first of its 13 articles on GT was published in 2000 and it continued with this line until 2018. It has the highest SJR impact with 0.972. Environmental Science and Pollution Research is the journal which is the most recent newcomer in this line of publication, with its first article published in 2015. However, in a brief period of time it has risen to the eighth position being one of the most prolific with eleven articles. It has an H index of 6, a total of 92 citations and an average of 8.4 citations per article. This German journal has an impact factor of 0.828 which places it in the first quartile of the SJR in the category of Environmental Sciences.

### 3.4. Most Relevant Countries in terms of Research in GT

A total of 83 countries participated in the research in GT during the whole period of study. However, only 34.9% of them have participated in at least five articles; while 30.1% have been involved in only one. On the other hand, there are enormous differences in this group of countries in terms of the level of economic and social development as well as geography and climate. All of this conditions the capacity of each country to research and publish studies on GT. Therefore, Table 3 shows the number of articles per million inhabitants of those countries that have published a minimum of 5 studies on GT, and the percentage that they represent of the total sample analysed. Furthermore, it includes the H index of the articles, the percentage of studies carried out through international collaboration, the total number of citations obtained and the average number of citations obtained by the articles carried out with and without international collaboration. 

On average, the number of articles per capita of the group of countries is 0.507. Eleven countries of the total 29 are above this average (Greece, Netherlands, Denmark, Spain, Israel, South Korea, Belgium, Sweden, Switzerland, Canada and Australia). With respect to the number of citations per article, eleven are above the average for the countries as a whole at 14.5 (Portugal, UK, Switzerland, Germany, Denmark, Spain, USA, France, Netherlands, Australia and Israel). With respect to the international collaboration in carrying out the studies, the group of 29 countries shows an average percentage of 36.7% of the total articles published. This percentage is lower compared to other related subjects [11,76,77,78,79]. Although a total of 16 countries are above this average, only eight reach at least 50% (UK, Germany, France, Mexico, Thailand, Australia, Israel, Malaysia). With respect to the number of citations obtained by the studies carried out with and without international collaboration with institutions of other countries, on average these two figures are balanced at 14.5 and 13.6 respectively. However, substantial differences may be observed between the countries. 

Greece is the country with the highest number of articles per capita with 1.958 articles per million inhabitants. With 2.97% of the articles of the sample it has accumulated a total of 270 citations which is 3.2% of the total of the sample. On average, these studies have 12.9 citations and an H index of ten. 38.1% of the studies have been carried out through collaboration with institutions of other countries. Its collaborators that account for the most number of studies are France and the Netherlands. In the studies that have been carried out through international collaboration an average of 16.1 citations has been obtained as opposed to the 10.9 of the rest of the studies. Greece’s first study on GT was published in 1999. There are three dominant themes in the GT research conducted in this country: greenhouse ventilation systems, climate control and biological pest control. In recent years, the development of automated systems and the use of sensors has been studied, particularly related to the afore-mentioned topics. 

The Netherlands has 1.625 articles per capita, representing 3.97% of the total. It has a total of 505 citations, representing 5.9% of the total and an H index of fourteen. 39.29% of its studies have been conducted through international collaboration, with Spain being the country with which it has most collaborated. The studies on technological development for climate control and the research on technology for ecological production systems particularly focused on horticultural crops are predominant. 

Denmark has 1.207 articles per capita, 0.99% of total studies. It has a total of 139 citations, representing 0.6% of the total. Its average number of references per article is 19.9 and its H index is six. 42.86% of its studies have been conducted through international collaboration, with the Netherlands being the country with which it has most collaborated. It has a higher average number of citations in its studies carried out through collaboration with international institutions. Prominent topics in the research carried out are climate control and the different aspects related to soil such as solarisation or technologies to eliminate heavy metals. 

Spain has 1.156 articles per capita. Its publications represent 7.65% of the total, the third highest percentage of all the countries. It accumulates a total of 1048 citations, representing 12.3% of the total. Its average number of citations per article is 19.4 and its H index is 21. Spain is one of the countries with the lowest percentage of articles published through international collaboration with only 18.5%. The countries with which it most collaborates are Mexico, the Netherlands and the UK. Spanish research has focused on technology to improve water efficiency, pest control and different aspects related to the optimum state of soil, such as the elimination of heavy metals, disinfection and soil amendment.

China has the highest percentage of articles of the sample with 34.28%; however, its number of articles per capita is only 0.174. The articles from this country have an average of 9.1 citations per article and an H index of twenty-six. 13.6% of Chinese articles are carried out through international collaboration with the USA being its principal collaborator. On average, China has a higher number of citations in these articles than those carried out alone. The Chinese research focuses on a more diverse range of topics as opposed to the high level of specialisation found in other countries.

Portugal is the country with the most number of citations per article, with 40.8. This country published 0.486 per million inhabitants, and represents 0.71% of the articles of the sample. It accumulates a total of 204 citations per article and has an H index of four. 20% of its studies are carried out through international collaboration with Brazil and the USA being its principal collaborators. 

### 3.5. Most Relevant Institutions in the Research in GT

Next, the most relevant characteristics of GT research are examined based on the institutions to which the authors of the studies analysed are affiliated. Table 4 includes the ten institutions with the highest number of articles of the sample analysed. These institutions are located in four different countries, with seven of them being in China. The institution with the highest number of articles is the Chinese Academy of Sciences with a total of 47. This is followed by the Ministry of Agriculture of the People’s Republic of China with 32 and the University of Almería of Spain with 26. With respect to the total number of citations, the Chinese Academy of Sciences is in first place with 578, followed by the University of Almeria with 522 and the Wageningen University and Research Centre of the Netherlands with 491. However, the latter has the highest average number of citations per article with 20.5, followed by the University of Almería with 20.1 and the Ministry of Education China with 17.4. 

With respect to the international collaboration of these institutions in carrying out their studies, in general they all have low percentages. The average percentage of joint collaboration of the group of the ten institutions is 18.1%. The Rural Development Administration of South Korea has a higher percentage of collaboration with 50.01%, obtaining a higher number of citations in its articles carried out in collaboration. Of its international collaborations, particularly noteworthy is the number of studies carried out with the Deutsche Sammlung von Mikroorganismen und Zellkulturen GmbH. The Wageningen University and Research Centre of the Netherlands has carried out 41.67% of its studies through international collaboration. Its partners include the University of Almería. However, this institution has obtained a lower average number of citations in its studies carried out in collaboration. The third institution with the highest percentage of collaboration, and the last that is above the average, is the Chinese Academy of Sciences with 21.28%. Its principal partners are a group of institutions from Australia, Germany and the USA. In this case, the studies carried out in collaboration obtain a higher acknowledgement in citations.

### 3.6. Most Relevant Authors in GT Research

**Figure 4:**
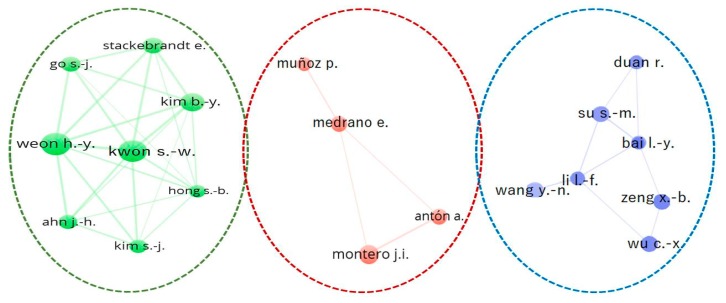
Main relationships between authors in GT research.

Table 5 shows the main characteristics of the authors with the highest number of articles on GT. This group of authors are affiliated to six different institutions in four countries. Only four of these authors have participated in at least 10 articles. Furthermore, the majority of these authors work in the same research group in the studies on GT. This is one of the reasons why the institutions to which they are affiliated are among the most prominent in terms of the number of articles. Figure 4 shows a graphic representation of the collaborative relationships established between the principal authors who participate in the publication of articles on GT.

The researcher with the most articles is Hang-yeon Weon, from the Rural Development Administration in South Korea, with a total of 20. This author accumulates a total of 346 citations, has an average of 17.3 citations per article and an H index of 12. The author with the second most articles is Soon-wo Kwon, from the same institution as the previous author, with 19 articles. This researcher has 335 citations, an average of 17.6 citations per article and an H index of 11. In third position is Byung-yong Kim, from the Chun Lab, Inc. in South Korea, with a total of 13 articles. This author has accumulated a total of 314 citations, has an average of 24.2 citations per article and has an H index of 11. The researcher with the highest average number of citations per article is Juan Ignacio Montero, of the Food and Agriculture Research and Technology Institute in Spain, with a total of 34.4. He has accumulated a total of 310 citations per article and has an H index of eight. Montero is also the most veteran author of the group in GT research with his first article in the sample being published in 1999. Jae-hyung Ahn and Soo-jin Kim, of the Rural Development Administration in South Korea are the most recent newcomers to this line of research with their first article on GT being published in 2013.

### 3.7. Principal Topics in GT Research 

The qualitative analysis of the sample of articles selected has enabled us to identify the most relevant topics in the research in GT. Subsequently, the quantitative analysis of the keywords used in the documents has allowed us to confirm the results previously obtained. In this way, we can confirm that the research in GT revolves around six topics: the use of water for irrigation, the design of the optimum structure of the greenhouse, conserving the soil in the best productive conditions, energy consumption of the system as a whole, climate control within the facility and pest control. These topics represent the principal limitations and consequences of protected agriculture to which technology seeks to provide a solution. Throughout the period of analysis (1999–2018) the concern about the afore-mentioned topics is maintained; however, there has been a significant evolution in the technology developed in each of the aspects. Although at the beginning of the period many studies focused on the distribution and design of lighting and ventilation systems, support of the structure, etc., currently there is a predominance of studies on automation systems, the development of sensors, the design of online applications, biological pest control, the genetic modification of crops, etc.

Another fundamental aspect which has been identified is the low level of collaboration between institutions, mainly due to the ad hoc development of the studies, designed for the study of a specific crop under specific conditions in specific regions. Therefore, the collaborations that have taken place have been driven by common interests with respect to the afore-mentioned aspects. The results show that an overall change of approach has taken place in the research in GT. Although at the beginning of the period, the principal motivation behind the majority of studies was the increase in production and cost reduction: currently, there has been a paradigmatic change in favour of more sustainable systems, from an economic, social and environmental point of view [39,64]. This is driven by different factors such as the demand pressure for more ecological and sustainably produced products, the greater awareness of the stakeholders about environmental aspects, the regulatory changes on an international level with respect to environmental conservation and clean production and the evidence of the consequences of climate change for the sector.

Finally, certain gaps in the literature on GT have been detected. On the one hand, the studies on GT are dominated by a technical approach. However, it is necessary to include economic analyses able to identify the economic and financial feasibility of the innovations for their widespread adoption by farmers [80]. We must not forget that, in many regions, the greenhouses are very small and their owners are small farmers with limited resources to purchase new equipment [81]. On the other hand, from a social perspective, it should be considered that it is not enough to develop highly efficient technology but the benefits of this technology must be appropriately transmitted to the final user. Therefore, it is necessary to examine further the factors that influence the adoption of technology by farmers, who are often reluctant to incorporate it [82]. The existing technological innovations are able to provide solutions to the environmental impacts derived from agricultural activity; however, soil and water pollution continues to be a problem in all regions of the world [36]. Therefore, more research is required on how regulation can drive the adoption of technological measures aimed at improving environmental conservation and the most efficient ways of funding the acquisition of technology to contribute to the sustainability of the agricultural activity [83]. 

**Figure 5:**
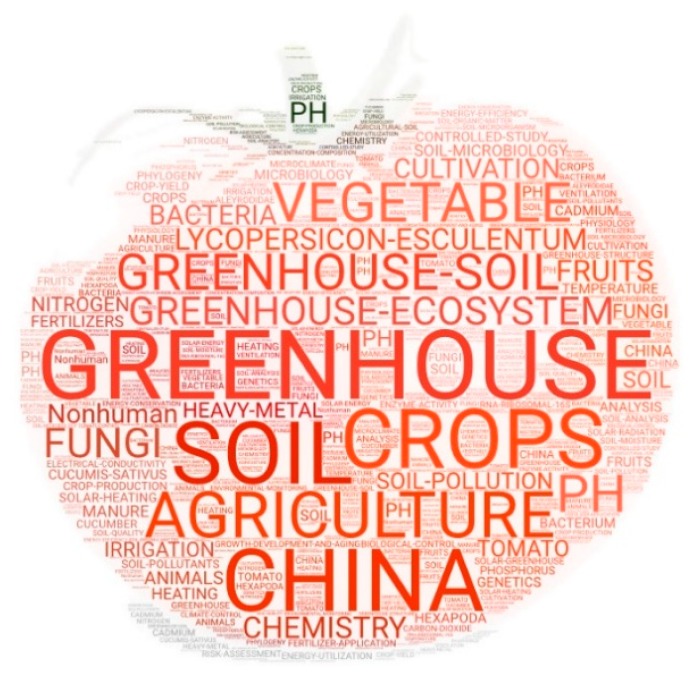
Word cloud based on the main keywords related to GT in worldwide research.

The keywords analysis of a sample of studies on a particular topic enable us to identify the research trends within the area of study [75,84]. Figure 5 shows a word cloud made up of the principal keywords gathered from the Scopus database for articles related to GT. Only those terms that appear in at least five articles have been included. As was expected “greenhouse” is the most representative term in the total articles that make up the sample, included in 42.2% of the articles. Other terms that include this word are “greenhouse-ecosystem”, “greenhouse-soil”, “greenhouse-structure”, “greenhouse-irrigation”, “greenhouse-climate”, etc. The second most used keyword is associated to one of the most relevant topics within the research on GT, “soil” with 19.7% of the total. The other most prominent keywords include general terms related to agricultural practices, such as “greenhouse-ecosystem” (11.6%), “agriculture” (10.6%), “vegetable” (8.5%), “crops” (7.1%), “cultivation” (6.9%). Among the 10 most used keywords is the term “Lycopersicon-Esculentum” with 9.6% of the total. This term is the botanic name of the solanaceae plant species, commonly known as tomato. This crop is the most representative within the research on GT. The term “tomato” is also prominent among the keywords with 5.1%. The second most representative crop is the “cucumber” with 3.1%, with the botanical name of the “Cucumis-Sativus” being particularly prominent with 5.1%. Also prominent but with much less repercussion are the terms referring to the species “Lactuca” (1.6%),the species of corn “Zea-mays” (1.6%), and “lettuce” (1.4%). With respect to the country where the studies or tests are carried out, “China” is prominent with 10.2%. With a much lower percentage, this is followed by “Spain” (1.8%), “South Korea” and “USA” (1.6%). 

**Figure 6:**
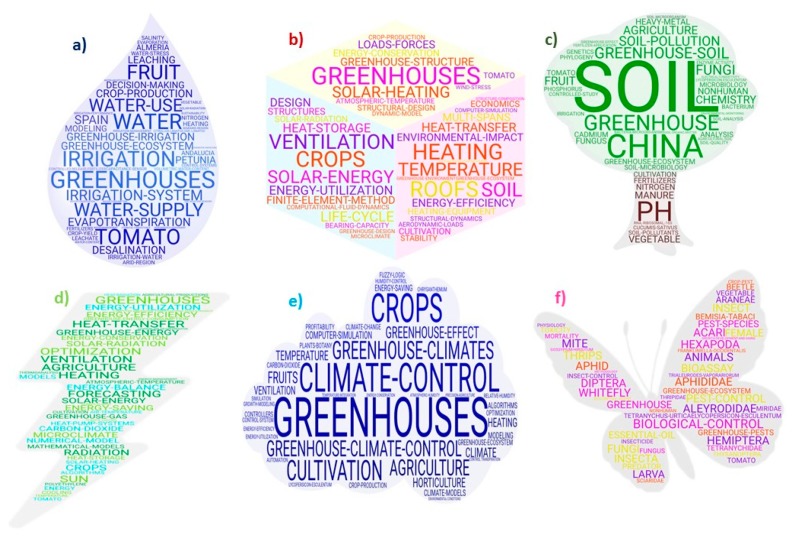
Word cloud based on the main keywords related to GT Irrigation (**a**), GT Structure (**b**), GT Soil (**c**), GT Energy (**d**), GT Climate (**e**), GT pest (**f**) research documents.

Figure 6 shows the different word clouds that include the principal keywords based on the six most prominent research topics in the field of GT. 

A. The GT research focused on irrigation is one of the dominant themes in the studies carried out in arid and semi-arid climates, where water is the principal limiting factor (Figure 6a). Spain, particularly the province of Almería, has carried out the majority of studies in this field. Among the most noteworthy aspects are the supply, use, consumption and quality of water, water stress and the resource conservation. The analysis of these studies includes a wide range of variables such as evaporation and evapotranspiration, soil humidity and water salinity. Efforts have focused on the development of irrigation systems aimed at improving the efficiency in the use of the resource, where automation, the use of sensors and the continuous control of the group of variables that affect water are under constant observation. Also noteworthy is the research in alternative sources of water for irrigation, either through centralised or decentralised measures (desalinated seawater, treatment of brackish water, reused water, rainwater) and the impacts of their use on the crops and the soil. 

B. The greenhouse structure has the objective of protecting the crops, particularly from the variable climate conditions in order to create a stable microclimate (Figure 6b). It is, therefore the support for all of the elements that intervene in the cultivation process (irrigation systems, heating, lighting, ventilation, etc.). This is reflected in the research in GT. The studies on structure can be divided into two groups: those focused on the structure itself (materials, distribution of posts, design); and those that analyse the structure as the support of some of the variable indicated (irrigation, heating, refrigeration, lighting, etc.). With respect to the first group of studies, the most relevant issues are the development of more resistant materials, the design of structures capable of withstanding the wind and the design of the structure adapted to the needs of the crop and focused on energy saving. Furthermore, in more recent years, larger structures are being developed in order to house fruit trees. In the second group we can find studies that relate the structure to the rest of the elements necessary for production, with an emphasis on solar heating, solar energy production systems, comprehensive pest control, etc. The studies on structure have mostly an economic approach, contrary to other studies. The models and tools used include the finite-element-method, computational-fluid-dynamics, life-cycle, computer-simulation, and the dynamic-model. 

C. With respect to the research related to the soil of the greenhouses, the studies focus on two basic issues: its conditioning for the optimum state for the crop and the remediation of pollution processes (Figure 6c). The country where the highest number of studies on this subject matter has been carried out is China. Particularly relevant fields in this group of studies are Chemistry, Genetics and Microbiology, contrary to the rest of the research topics. With respect to methodology, controlled studies, monitory and soil analyses are most prominent. Fertilization is given special attention as are soil disinfection processes. The most salient keywords include: soil-pollution, heavy-metal, bacteria-(microorganisms), soil-pollutants, ph, fungi, nitrogen, manure, phosphorus, rna-ribosomal-16s, cadmium, enzyme-activity, soil-microorganism, phylogeny, bacterium, soil-organic-matter, fungus.

D. E. The heating systems are the main energy consumers in those facilities that use them. This is why the research on energy consumption is closely related to climate control (Figure 6d,e respectively). In the studies on the use of energy, there are three basic issues: the efficient use of energy and energy saving, the use of systems that include renewable energy and climate control as the principal energy consumer. With respect to methodology, the numerical-model and mathematical-models, the use of algorithms, computational-fluid-dynamics, computer-simulation are noteworthy. In the studies on climate inside the greenhouse one of the prominent themes is the use of energy and energy efficiency. But, within this theme, we can also find a wide group of issues such as heating, refrigeration, ventilation or humidity that comprise the microclimate of the facility. This subject area has placed the most attention on climate change, particularly with regard to the increase in temperatures. The methodologies that are most referred to are computer-simulation, climate-models, control-system, controllers, growth-modelling, algorithms, modelling, fuzzy-logic, simulation, automation.

F. The final priority topic in GT research is pest control (Figure 6f). Although it is not directly related to the structure of the greenhouse per se, it is considered appropriate to include pest control here as it is fundamental for the development of the activity. The evolution of market trends in recent years has given rise to international regulations regarding pest control and the use of products that have harmful effects on health. Therefore, the technology has had to face the challenge of protecting the crops from pests that evolve with climate change, using systems that respect the environment and that are innocuous for health. As a result, biological control has become the preferred alternative. The fields of Biology and Genetics are making huge steps, particularly in the development of resistant varieties and complementarities between different species. The most used keywords include the terms referring to the principal threats and predators: aleyrodidae, hexapoda, animals, acari, hemiptera, pest-species, aphididae, bemisia-tabaci, diptera, frankliniella-occidentalis, tetranychidae, fungi, aphid, tetranychus-urticae, whitefly, insect, thrips, trialeurodes-vaporariorum, thysanoptera, araneae, mite, predator, beetle, sciaridae, miridae, thripidae, fungus.

## 4. Conclusions

The objective of this study has been to reveal the current status and evolution of the research in greenhouse technology and its areas of application. It has identified the principal driving agents of the research in this subject, the most prominent lines of research and the technology-intensive areas and gaps in the research. 

The sustainable intensification of agriculture is one of the most plausible alternatives to respond to the challenge of food supply in the twenty-first century and greenhouse farming has been proved to be efficient over the last few decades. Technology has evolved to optimize greenhouse production systems, particularly with regard to irrigation, greenhouse structure, soil, energy use, climate control and pest control. 

Research in GT has been characterised by responding to clearly contextualised problems for a type of crop in a specific region with a particular climate, a specific resource endowment, within a legislative framework that conditions the use of products (fertilizers, herbicides, pesticides) and which serves a global market. This conditions the international relationships between institutions and authors of the research in GT. 

The evolution of consumption patterns and the degree of social awareness has given rise to what we could call a change of the paradigm in the research in GT. Although the agricultural activity is developed by entrepreneurs and their interests must include the maximisation of productivity and the search for profit, agriculture has experienced a process of incorporating values related to sustainability. Therefore, resource conservation—particularly water and soil - and social acceptance are currently priority concerns within the sector. 

In response to the new challenges of the sector, the research in GT should address issues such as the economic-financial feasibility of the innovations for their widespread adoption by farmers; communication from the scientific field with the rest of the interested parties, both to transmit the results obtained and to give feedback regarding the development of projects focused on providing solutions for the needs of the sector; and to secure the participation of the institutions and governments to jointly respond to the different problems that exist. Moreover, there is a considerable gap between the scientific knowledge and the end-users’ believes regarding technology. For this reason, it is important to correctly transfer the developed technological advances to the end-users. In this way, efforts should be made to achieve sustainability, including the economic, social and environmental fields.

## Figures and Tables

**Table 1:** Major characteristics of greenhouse technology (GT) research.

Year	Articles	Authors	Journals	Countries	Citation	Average Citation ^1^
1999	11	32	10	11	1	0.1
2000	8	21	8	7	6	0.4
2001	7	19	7	6	19	1.0
2002	17	50	16	12	22	1.1
2003	19	54	18	13	24	1.2
2004	19	64	17	11	59	1.6
2005	24	77	20	13	104	2.2
2006	30	95	21	16	148	2.8
2007	27	99	22	16	211	3.7
2008	19	84	18	13	266	4.8
2009	36	140	26	19	312	5.4
2010	36	157	29	20	379	6.1
2011	48	194	41	22	448	6.6
2012	44	189	32	27	563	7.4
2013	53	239	41	25	643	8.1
2014	55	238	43	23	721	8.7
2015	54	229	40	19	838	9.4
2016	63	312	48	25	988	10.1
2017	71	287	61	29	1236	10.9
2018	67	319	51	27	1541	12.1

^1^ Total number of citations accumulated to date divided by the total number of articles published to date.

**Table 2:** Major characteristics of the most active journals related to GT research.

Journal	Articles	SJR ^1^	H Index ^2^	Country	Citation	Average Citation ^3^	1st Article	Last Article
Nongye Gongcheng Xuebao Transactions of the Chinese Society of Agricultural Engineering	49	0.422 (Q2)	10	China	309	6.3	2005	2018
Acta Horticulturae	30	0.185 (Q4)	5	Belgium	49	1.6	1999	2018
International Journal of Systematic and Evolutionary Microbiology	17	0.912 (Q1)	11	UK	327	19.2	2006	2016
Biosystems Engineering	15	0.834 (Q1)	11	USA	323	21.5	2002	2018
Chinese Journal of Applied Ecology	14	0.267 (Q4)	4	China	70	5.1	2004	2018
Hortscience	14	0.424 (Q2)	6	USA	185	13.2	2001	2017
Biological Control	13	0.972 (Q1)	10	USA	444	34.2	2000	2018
Environmental Science and Pollution Research	11	0.828 (Q1)	6	Germany	92	8.4	2015	2018
Journal of Food Agriculture and Environment	10	0.132 (Q4)	4	Finland	25	2.5	2008	2017
Pedosphere	10	0.952 (Q1)	8	China	130	13.1	2001	2017

^1^ Scimago Journal Rank 2018 (Based on the category of Agricultural and Biological Sciences, except Environmental Sciences and Pollution Research that is based on Environmental Sciences); ^2^ Only sample documents; ^3^ Total number of citations divided by the total number of articles.

**Table 3:** Main characteristics of the most active countries related to GT research.

Country	Average per Capita Articles ^1^	Percentage of Total Articles	H Index ^2^	Percentage of Collaboration ^3^	Citation	Average Citation ^4^	Average Citation	
							Collaboration ^5^	Non Collaboration ^6^
Greece	1.958	2.97	10	38.10	270	12.9	16.1	10.9
Netherlands	1.625	3.97	14	39.29	505	18.0	14.4	20.4
Denmark	1.207	0.99	6	42.86	139	19.9	27.7	14.0
Spain	1.156	7.65	21	18.52	1048	19.4	18.0	19.7
Israel	1.126	1.42	7	50.00	161	16.1	15.0	17.2
South Korea	0.910	6.66	18	38.30	648	13.8	26.4	5.9
Belgium	0.700	1.13	5	12.50	88	11.0	3.0	12.1
Sweden	0.589	0.85	4	33.33	64	10.7	10.5	10.8
Switzerland	0.587	0.71	5	40.00	163	32.6	21.5	40.0
Canada	0.567	2.97	10	33.33	250	11.9	9.3	13.2
Australia	0.560	1.98	9	50.00	232	16.6	13.1	20.0
Portugal	0.486	0.71	4	20.00	204	40.8	5.0	49.8
Germany	0.362	4.25	17	70.00	665	22.2	27.4	10.0
Turkey	0.352	4.11	9	13.79	399	13.8	9.8	14.4
Malaysia	0.317	1.42	4	50.00	55	5.5	6.8	4.2
Italy	0.314	2.69	9	10.53	273	14.4	40.5	11.3
USA	0.300	13.88	24	40.82	1839	18.8	22.6	16.1
Romania	0.257	0.71	2	20.00	29	5.8	1.0	7.0
UK	0.226	2.12	9	86.67	504	33.6	37.2	10.0
Japan	0.213	3.82	8	40.74	178	6.6	9.5	4.6
Iran	0.196	2.27	7	18.75	73	4.6	2.3	5.1
China	0.174	34.28	26	13.64	2182	9.0	19.6	7.3
France	0.149	1.42	4	70.00	186	18.6	11.9	34.3
Thailand	0.101	0.99	3	57.14	75	10.7	17.8	1.3
Colombia	0.101	0.71	3	20.00	5	1.0	0.0	1.3
Brazil	0.072	2.12	7	13.33	72	4.8	2.5	5.2
Mexico	0.063	1.13	5	62.50	97	12.1	8.2	18.7
Russia	0.035	0.71	2	40.00	25	5.0	10.0	1.7
India	0.011	2.12	7	20.00	137	9.1	14.3	7.8

^1^ Total number of articles per million inhabitants; ^2^ Only sample documents; ^3^ Number of articles made through international collaboration divided by the total number of articles; ^4^ Total number of citations divided by the total number of articles; ^5^ Number of citations obtained by articles made through international collaboration divided by the number of articles; ^6^ Number of citations obtained for articles not made through international collaboration divided by the number of articles.

**Table 4:** Main characteristics of the most active institutions related to GT research.

Institution	Country	Articles	Citation	Average Citation ^1^	H Index ^2^	Percentage of Collaboration ^3^	Average citation	
							Collaboration ^4^	Non Collaboration ^5^
Chinese Academy of Sciences	China	47	578	12.3	15	21.28	25.2	8.8
Ministry of Agriculture of the People’s Republic of China	China	32	235	7.3	9	6.25	14.5	6.9
University of Almeria	Spain	26	522	20.1	11	15.38	11.8	21.6
China Agricultural University	China	25	135	5.4	7	16.00	8.5	4.8
Wageningen University and Research Centre	Netherlands	24	491	20.5	13	41.67	15.6	23.9
Rural Development Administration	South Korea	24	356	14.8	12	50.01	25.1	4.6
Chinese Academy of Agricultural Sciences	China	20	170	8.5	8	0.00	0.0	8.5
Ministry of Education China	China	17	296	17.4	7	17.65	69.0	6.4
Zhejiang University	China	17	243	14.3	7	11.76	16.0	14.1
Shenyang Agricultural University	China	16	70	4.4	6	0.00	0.0	4.4

^1^ Total number of citations divided by the total number of articles; ^2^ Only sample documents; ^3^ Number of articles made through international collaboration divided by the total number of articles; ^4^ Number of citations obtained by articles made through international collaboration divided by the number of articles; ^5^ Number of citations obtained for articles not made through international collaboration divided by the number of articles.

**Table 5:** Major characteristics of the most active authors related to GT research.

Author	Articles	Citation	Average Citation ^1^	H Index ^2^	Country	Affiliation ^3^	1st Article	Last Article
Weon, Hang-yeon	20	346	17.3	12	South Korea	Rural Development Administration	2006	2016
Kwon, Soon-wo	19	335	17.6	11	South Korea	Rural Development Administration	2006	2016
Kim, Byung-yong	13	314	24.2	11	South Korea	Chun Lab, Inc.	2006	2014
Stackebrandt, Erko	10	288	28.8	10	Germany	Deutsche Sammlung von Mikroorganismen und Zellkulturen GmbH	2006	2007
Go, Seung-joo	9	254	28.2	9	South Korea	Korean Agricultural Culture Collection	2006	2007
Montero, Juan Ignacio	9	310	34.4	8	Spain	Institut de Recerca I Technologia Agroalimentaries	1999	2018
Ahn, Jae-hyung	8	35	4.4	4	South Korea	Rural Development Administration	2013	2016
Bai, Lingyu	7	78	11.1	4	China	Ministry of Agriculture of the People’s Republic of China	2009	2018
Kim, Soo-jin	7	25	3.6	4	South Korea	Rural Development Administration	2013	2016
Li, Lianfang	7	73	10.4	4	China	Ministry of Agriculture of the People’s Republic of China	2009	2018

^1^ Total number of citations divided by the total number of articles; ^2^ Only sample documents; ^3^ Last verified affiliation.
